# Now You Do It, Now You Don’t: The Mixed Blessing of Creative Deviance as a Prosocial Behavior

**DOI:** 10.3389/fpsyg.2020.00313

**Published:** 2020-03-05

**Authors:** Jigyashu Shukla, Ronit Kark

**Affiliations:** ^1^University of Central Florida, Orlando, FL, United States; ^2^Department of Psychology, Bar-Ilan University, Ramat Gan, Israel; ^3^Business School, University of Exeter, Exeter, United Kingdom

**Keywords:** prosocial behavior, creative deviance, creativity, resources, LMX

## Abstract

Creative deviance, the act of developing an idea by an employee even when it was banned by the manager, is a novel and interesting construct that can bring both positive and negative outcomes to organizations. The construct of creative deviance is neglected in the existing literature and the theory development for creative deviance is still in the nascent stages. We expand the theoretical nomological network of creative deviance by introducing prosocial motivation as an antecedent of creative deviance and developing a multilevel model of the moderators of this relationship. Creative deviance can occur due to pro-self or prosocial intentions. In our paper, we focus on the prosocial intentions behind acts of creative deviance. We illustrate how the prosocial motivation can lead to creative deviance and how creative deviance in turn, can act as a double-edged sword leading to positive outcomes of creative performance and innovation as well as negative outcomes of wastage of resources and deteriorated leader member exchange. Our model delineates the boundary conditions influencing the relationship between creative deviance and its outcomes. Specifically, we explore the theoretical foundations of social skills and perspective taking, as the individual level moderators; team network structure and climate of excellence, as moderators at team level; organizational structure at organization level; and uncertainty as the external environmental level moderator.

## Introduction

‘My incentive was saving human lives,’ says Brig. Gen. Daniel Gold, who defied the defense establishment to forge ahead with Iron Dome in 2005. The Israeli defense establishment thought Brig. Gen. Daniel Gold felt like Don Quixote and was absolutely crazy when he broached the idea for the missile-defense system that came to be known as Iron Dome (Kippat Barzel in Hebrew). Several years later, Iron Dome [while its development was not fully authorized by the military and the effort to keep working on it without full permission of all ranks was critiqued by the Israeli State Comptroller], turned out to be the surprise hero of the 2012 and 2014 wars and military operation saving the lives of many civilians and protecting population centers against missiles. In 2012 Gold won the Israel Defense Prize in 2012 for spearheading the Iron Dome project. -News article in Israel21c (Leichman, 2014).

There are many cases in organizations in which employees disobey the requests of supervisors to stop working on a creative solution to a problem or on a project with creative potential. Such incidents come under the conceptual umbrella of “Creative Deviance,” a relatively new construct. Mainemelis (2010) first delineated the theoretical foundations of creative deviance and defined it as “*the violation of a managerial order to stop working on a new idea*.” Creative deviance has two major components: the deviant behavior, which is usually seen as a negative behavior in organizations, as well as the “creative” element, which is mostly seen positively and can contribute to organizational performance and growth. Thus, creative deviance is a unique and complex deviant behavior, in comparison to other deviant organizational behaviors. For example, insulting or mistreatment of coworkers, sabotaging regulatory property, missing work-hours, and other similar behaviors fall under the category of destructive deviance, and all lead to negative outcomes whereas creative deviance can lead to both negative and positive outcomes (Mainemelis, 2010).

Lin et al. (2016) studied leaders’ responses to followers’ creative deviance. They established that the different responses to this disobedient behavior (e.g., punishment, reward, forgiveness) can lead to different outcomes in the long-term, such as enhancing creativity and innovation or subsequent creative deviance. There are various positive reasons for which followers may decide to enact creative deviance. As evident in the example of Brig. Gen. Daniel Gold, who aimed to save people’s lives, one’s motive can be ideological. Other reasons include affective commitment toward the organization, with innovation as a core value or motivation to give the client and customer the best product and service possible. However, there can be negative reasons for enacting creative deviance as well, such as using organizational resources for a self-focused, narcissistic, self-enhancing endeavor, without aiming to contribute to the organization or to the wider society. When employees seek to benefit others, such as external stakeholders, other organizational members, or the overall organizational performance, their prosocial motivation can lead and urge them to deviate creatively.

In the current paper, we develop a conceptual framework that aims to shed light on the prosocial motivation as an antecedents of creative deviance. We attempt to understand the boundary conditions of when creative deviance is likely to lead to positive outcomes, and when it can result in adverse outcomes. The novel construct of creative deviance, which has received limited attention in the extant literature, is vital because it is evident in the field, touches on a significant stage in the creative process, and can predict various positive and negative organizational outcomes. These outcomes contribute to creativity and innovation, or, in other cases, lead to wastage of various important resources (e.g., time, money, loss of materials, and reputation).

In this paper, our aims are threefold. We first aim to theoretically demonstrate why creative deviance may be enacted due to pro-social motivation. Until now, creative deviance has not been studied through a prosocial lens. The ability to link prosocial literature with that of creative deviance is valuable since deviant behavior can have prosocial motives (Umphress et al., 2010). We apply the motivated information processing theory (Kunda, 1990; Nickerson, 1998) to support our arguments establishing the relationship between prosocial motivation and creative deviance. Our second goal is to explore different types of outcomes (positive and negative) of creative deviance, demonstrating that prosocially motivated creative deviance can be a mixed blessing to organizations and can lead to both positive and negative outcomes. Our third aim is to suggest boundary conditions at varying levels of analyses, that moderate the relationship between creative deviance and its consequences. Specifically, drawing on the interactionist perspective of creativity (Woodman and Schoenfeldt, 1990; Woodman et al., 1993), we propose conditions under which prosocially motivated creative deviance is likely to result in positive outcomes, or in outcomes that may harm the organization. Hence, we provide theoretical foundations for a multi-level model that outlines factors (at four different levels, i.e., individual, team, organizational and environmental) which can influence the relationship between creative deviance and its outcomes.

For each level, we chose relevant exemplary boundary conditions, based on the interactionist perspective of creativity. At the ***individual level***, we focus on individuals’ *social skills* and *perspective taking* as the moderators of the relationship between creative deviance and its outcomes. At the ***relational/team level***, we focus on the *team network* structure and *team climate* as moderators. At the ***organizational level***, we explore the impact of the *organizational structure.* Lastly, at the broader ***external environment level*,** we focus on the *uncertainty in the external environment* as a meaningful moderator of the relationship between creative deviance and its outcomes.

**FIGURE 1:**
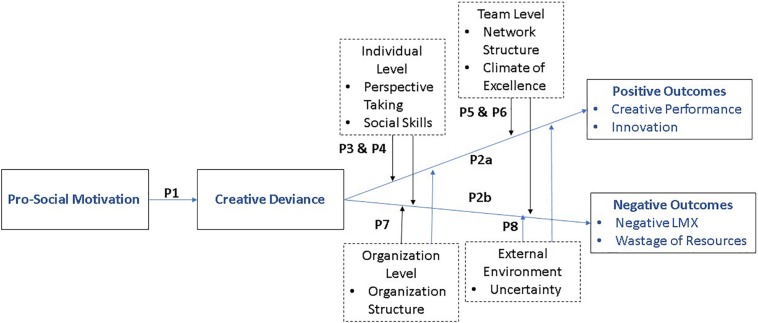
Theoretical model of creative deviance mediating prosocial motivation to positive/negative outcomes.

This theoretical framework can help us gain insight into how prosocial motivations can result in creative deviance, and how creative deviance can be a mechanism linking prosocial motivation to harmful consequences for an organization. It can further contribute to the field of prosocial behavior by enhancing our understanding of the double-edged sword effect of creative deviance ensued by prosocial motivation, as well as furthering our understanding of the multi-level conditions which can promote or hinder organizational outcomes of creative deviance. Below we define and link prosocial motivation to creatively deviant behaviors, then develop and present our multi-level conceptual framework. Our overall model is shown in Figure 1.

## Prosocial Motivation and Creative Deviance

Creative deviance can be enacted because of either pro-self or prosocial motivations. The construct of ‘creative deviance’ links two different aspects, with somewhat different valence attached to each. The first is deviant behavior, which is perceived as involving a self-serving and pro-self-orientation (e.g., Wells, 1978; Pletzer et al., 2018), and the second is creativity, which although can be pro-self-motivated, has mostly been noted to involve an other/prosocial orientation (e.g., Kováè, 1998; Grant and Berry, 2011; Chou et al., 2013; Mouchiroud and Zenasni, 2013; Kemmelmeier and Walton, 2016; Amabile, 2018). Thus, the linked construct of ‘creative deviance’ can have both prosocial and pro-self antecedents. The prosocial behaviors and dispositions, including altruism (Rushton et al., 1981), extra role behaviors (Van Dyne and LePine, 1998) and more, can have a positive relationship with creative deviance. Similarly, pro-self behavior and dispositions, including narcissism (obsession with the idea of oneself), territoriality for an idea (Brown et al., 2005), and more, can lead to creative deviance. Nonetheless, in the current theoretical framework, we focus on understanding creative deviance that is enacted due to prosocial motivation. We focus on the prosocial motivation to creative deviance for two reasons:

First, little is known about the possible negative outcomes of the prosocial behavior. Pro-self behavior is connected to the negative outcomes such as low social responsibility, low reciprocity, maximizing own outcomes (De Cremer and Van Lange, 2001; Eek and Garling, 2008) and positive outcomes such as value claiming, career commitment, and proactivity (Den Hartog and Belschak, 2007; Belschak and Den Hartog, 2010; Brett and Thompson, 2016). Whereas pro-self behavior can have positive as well as negative outcomes, the existing literature is focused majorly on the positive components of prosocial behavior (Belschak and Den Hartog, 2010; Brett and Thompson, 2016) except some limited research on resource depletion as a negative outcome of prosocial behavior (Bartlett and DeSteno, 2006). Thus, it is of importance to gain a deeper understanding of the possible negative outcomes of prosocial motivation, since prosocial behavior is encouraged in organizations without considering its possible negative side, which can in turn lead to counterproductive human resource strategies. In order to contribute to this limited area of research and at the same time develop the nomological network of creative deviance(a novel construct that has not yet received a lot of research attention), we explore how creative deviance may be derived from prosocial motivation and when it can lead to both positive and negative outcomes. Our focus on prosocial motivation is in accord with the theme of this issue.

Second, pro-self motivation leading to other outcomes (guided by boundary conditions) through creative deviance is a separate model. It is important to note that pro-self and prosocial motivation can lead to the different outcomes through creative deviance. There can be various outcomes such as low learning orientation (Wales et al., 2013), process conflict (Behfar et al., 2011) and others which can be specifically linked to pro-self antecedents through creative deviance. However, to discuss both prosocial and pro-self based antecedents and their respective outcomes in depth is beyond the scope and space limitations of this article. In the current article, we focus on prosocial motivations and how it leads to positive as well as negative outcomes through creative deviance.

Prosocial motivation is signified by strong intent to make a positive difference in the lives of others (Batson, 1987; Grant, 2007). When individuals are high on prosocial motivation, they will go beyond the call of duty to make a difference in the lives of others (Grant, 2007, 2008). The extant literature has established many positive outcomes of prosocial motivation. For example, prosocial motivation fuels passion for the cause, which in turn prompts individuals to act within their organization, thus leading to perseverance, better performance, and greater efficiency (Thompson and Bunderson, 2003). Prosocial motivation contributes to the development of a basic moral sense amongst employees and enables them to create personal values related to their jobs (Shamir, 1990). Prosocial motivation creates a desire to act on negative feedback as well as improve upon the work’s quality (Meglino and Korsgaard, 2004). “Commitment toward perceived beneficiaries” is the mechanism proposed to underlie the positive outcomes of prosocial motivation (O’Reilly and Chatman, 1986).

According to this perspective, two major psychological processes are triggered among employees that have a strong prosocial motivation: (a) the employee becomes aware of the impact of his/her action on the beneficiaries and (b) the employee feels an affective commitment toward the beneficiaries (Grant, 2007). In other words, employees who have prosocial motivation, feed the purpose behind their actions by contemplating the impact of their actions (on respective beneficiaries) in their respective roles, and feel a strong desire to care for the potential beneficiary (Grant, 2007). Affective commitment refers to the emotional concern an employee feels toward the respective beneficiary of prosocial motivation. In many cases, the above recipients can be internal (e.g., managers and co-workers) or external stakeholders (e.g., customers, collaborators from linked organizations, and others), as well as the wider society. For example, an employee may be involved in a project in which he or she interacts directly with the customer, and due to prosocial motivation to add value for the customer, a strong interpersonal relationship can be formed. There are many studies that have explored the formation of strong interpersonal bonds between employees of an organization and respective “customers,” examples being those bonds between teachers and students (Ashton and Webb, 1986), between advocates and clients (Mann, 2004), and between nurses and their patients (O’Baugh et al., 2003).

The existing literature provides enough evidence that employees go beyond social norms with intent to benefit the customers, organizations, and co-workers (Morrison, 2006; Dahling et al., 2012). In the current paper, we suggest that these strong interpersonal relationships with other stakeholders and commitment to a cause (e.g., having a healthier environment and eco-system) – within the organization and outside the organization – motivate the individuals to go beyond the norm of saying “yes” to supervisor. This affective commitment toward other stakeholders and ideals will drive the employees to implement creative solutions to the problems faced by stakeholders such as customers, other managers (as opposed to focal employees’ personal supervisors), focal employees’ project collaborators, as well as the wider society.

In the context of creativity, existing research shows that prosocial motivation can lead to the development and implementation of creative ideas for the beneficiaries (see, for example, McAdams and de St Aubin, 1992; Grant and Berry, 2011). What would happen if the supervisor of the focal employee orders him/her to stop working on such creative idea? That might not stop the employee from continuing to work on the project because of the intense desire to make a “prosocial difference.” The reason for that disobedient behavior would be the strong affective commitment toward the other stakeholder – a mode of commitment, which has been found to increase with the amount of time and effort an employee devotes to the creative project (Allen and Meyer, 1990).

Employees would be ready to ignore the directions of a supervisor if they have already invested time and effort in their creative project, as well as in their attempt to reward the other stakeholders with whom they have relationships. This disobedience would be to avoid the cognitive consequences of negative emotions for the employee, such as regret for the sunk cost or regret of betraying the organization’s stakeholders, by not acting on an idea he or she thinks is brilliant, and that may be successful. The tendency to avoid those feelings would lead employees to stay engaged and invest in the original banned idea; despite the possibility of unfavorable consequences for the risk involved (Staw, 1976). Thus, the prosocial motivation to help some focal stakeholders, as well as to promote the general wellness and better performance of the organization will generate affective commitment, enabling an employee to ignore the hazards of neglecting another stakeholder (i.e., the supervisor).

Furthermore, if the cause is important – such as saving lives or coming up with an invention that will facilitate general wellness – a person with prosocial motivations will likely invest in the cause to help better the social good, at the expense of not complying with both the firm and the manager. The “motivated information processing theory” (Kunda, 1990; Nickerson, 1998; Grant and Berry, 2011) supports this argument, as it suggests that individuals recognize, ruminate and absorb only those pieces of social information which are compatible with their own motivation. Thus, employees with strong prosocial motivation will pay attention to the demands of targeted beneficiaries and generate innovative solutions. The manager’s orders, which are incongruent with the employees’ prosocial motivation, might be ignored in these situations, contributing to acts of creative deviance.

Our assertion is also supported by previous research, which has established that employees ignore the expectations of one stakeholder if they have a robust prosocial motivation to add value to other stakeholders. The literature on multiple commitments at the workplace (Reichers, 1986; Gregersen, 1993; Cohen, 2003, 2006) suggests that employees may favor commitment toward one stakeholder over another. The prosocial motivation, leading to choose one commitment over other, can become an antecedent of creative deviance. For example, Bendell (2017) found that the prosocial motivation (targeted toward the society and customers) of top management would lead to their commitment to both the social and customer objectives. To satisfy the customer and the social objectives, they would reject the innovative environmental practices that are expected by other stakeholders, like the government.

Similarly, an individual’s urge to make a prosocial difference in his/her organization might drive him/her to ignore the established moral norms of society, and he/she might engage in unethical pro-organizational behavior (Umphress et al., 2010; Umphress and Bingham, 2011). More recently, Thau et al. (2015) showed that the prosocial motivation targeted toward an individual’s group might drive the individual to become involved in pro-group, unethical behavior. Specifically, the prosocial desire to contribute to the well-being and performance of the group was positively related to the tendency to neglect the moral norms established by the organization or society.

The above research studies provide strong evidence that the strength of the prosocial motivation to add value to some target stakeholders, drives the focal individual to ignore the expectations of other stakeholders – such as one’s supervisor – and to possibly deviate. The reason for this deviance is the commitment toward the supervisory mandate (when the supervisor asks an employee to stop working on the project related to the benefit of the respective stakeholders) is in counter position to the commitment toward the stakeholders who are beneficiaries of a creative project. Among those opposing commitments, the focal employee might choose the commitment toward beneficiaries of the creative project. Thus, multiple commitments (Reichers, 1986; Gregersen, 1993; Cohen, 2003, 2006) in the workplace is a mechanism due to which, employees who are high on prosocial motivation to add value to organizations’ stakeholders, keep working on the creative idea and ignore the mandate of the supervisor. We propose that:

*Proposition 1*: Prosocial motivation is positively associated with creative deviance.

## Outcomes of Creative Deviance

Mainemelis (2010) outlined the organizational level predictors and boundary conditions of creative deviance. We expand the theoretical nomological network of creative deviance by exploring the consequences of prosocially motivated creative deviance. We argue that creative deviance, which is prosocially motivated, can still be a double-edged sword. It can lead to both positive and performance-enhancing individual outcomes, as well as negative and hampering ones. More specifically, focusing on creative deviance research, we explore the positive outcomes of creative deviance in the forms of creative performance and innovation. We also explore the negative outcomes of creative deviance, such as the waste of organizational resources (e.g., time, money, and reputation) or deteriorated leader-member exchange (LMX) due to tension in supervisor-subordinate relationships. We delineate the effects of creative performance on the positive and negative outcomes in more detail in the upcoming section.

### Creative Deviance and Positive Outcomes

*Creative performance* is defined as the product of an employee’s work that his or her manager views as novel and useful (Amabile et al., 1996; Zhou and George, 2001; Shin et al., 2012). In other words, creative performance is perceived as not only an original idea, but rather one that has potential to lead to new products, which are able to be utilized successfully by concerned stakeholders. *Innovation* has much overlap with the construct of creative performance. However, there are subtle differences between innovation and creativity. In order to clarify the distinction between creativity and innovation, we quote Anderson et al. (2014): “*Creativity and innovation at work are the process, outcomes, and products of attempts to develop and introduce new and improved ways of doing things. The creativity stage of this process refers to idea generation, and innovation refers to the subsequent stage of implementing ideas toward better procedures, practices, or products. Creativity and innovation can occur at the level of the individual, work team, organization, or at more than one of these levels combined, but will invariably result in identifiable benefits at one or more of these levels of analysis*.”

It is important to note that we are concerned with the individual level innovation in this article. The above definition suggests that innovation is more about the *implementation* of the new idea, whereas creative performance is more about the *generation and development* of the new idea at the individual level. Since creative deviance aids the generation and implementation of the new idea, we argue that creative deviance can lead to better creative performance as well as to innovation at the individual level. Below we explain how creative deviance will relate to these positive outcomes.

First, although an act of creative deviance can lead to waste and be counterproductive in a particular context, contexts are dynamic and may change; as the context changes in due course of time, the same product can become useful. In other words, creative deviance increases the likelihood that the idea will result in a product that the decision makers in the organization of the focal employee will accept as novel and useful at a later stage (Mainemelis, 2010). Although creative deviance is uncertain in its outcomes, it gives employees a “second chance” to pursue new ideas that they otherwise would have had to abandon. This “second chance” can lead to a better solution to an existing organizational problem since the employee gains more knowledge, learning, and skills while working on a specific idea. Thus, over a period, creative deviance can lead to creative problem-solving and subsequently innovation at the individual level.

The second way in which creative deviance can contribute to creativity and innovation is through the indirect autonomy the focal employee gains by indulging in creative deviance. Prior research has shown that autonomy is an essential stimulant of employee creativity (Amabile et al., 1996; Shalley et al., 2004; Anderson et al., 2014). Autonomy is manifested in the freedom in the day-to-day conduct of one’s work and freedom in deciding how to achieve the overall goal or mission of a project, which induces “a sense of control” over one’s own work and ideas” (Amabile, 1988). When a manager instructs an employee to stop working on a new idea, they limit the employees’ autonomy to explore the idea further. When an employee violates the managerial order, he or she illegitimately reclaims the autonomy to develop the new idea further (Mainemelis, 2010). In this way, creative deviance gives the focal employee a greater “strategic autonomy” (Criscuolo et al., 2013) to engage in the creative process while experiencing freedom from direct managerial control. This autonomy empowers the focal employee and can enhance his motivation to develop and implement innovative products and ideas.

The third way is the protection of the idea from undue criticism from the supervisor until the idea bears fruit. According to Staw (1990), managers often reject new ideas due to premature evaluation. Creative deviance allows employees to delay these types of early assessment of their ideas by managers. By engaging in creative deviance, employees gain the ability to further explore their ideas without having to justify their processes, following organizational procedures and documentation, or regulating their ideas before they are reasonably well developed. Thus, they avoid the trap of premature exposure of an idea before it is “ripe” for organizational use (Criscuolo et al., 2013), and do not need to generate evidence about the feasibility and utility of these ideas for their organization in initial stages (Augsdorfer, 2005; Mainemelis, 2010). Such an advantage can allow employees a more extended period of idea elaboration in which they can refine their creatively deviant ideas, leading to better creativity and innovation by the focal employee.

Fourth, creative deviance can strengthen intrinsic motivation against extrinsic motivation. Intrinsic motivation facilitates innovation and creative performance, whereas extrinsic motivation leads to job performance based on reward systems (Gagné and Deci, 2005; Grant and Berry, 2011). Authorized, expected and rewarded behavior can decrease intrinsic motivation (Deci et al., 1999) creativity (Amabile et al., 1996; Liu et al., 2016), and creative performance (Deci et al., 1999). Creativity has been associated with inner-directed and means-orientated behavior and focuses on positive challenges (Amabile, 1988; Zhang and Bartol, 2010; Liu et al., 2016). Creative deviance is a non-authorized, non-expected and risky behavior that is supported by high levels of intrinsic motivation and passion that an employee might have for the idea that he or she is working on (Liu et al., 2016). In having a sense of heightened control over their actions as well as secure emotional attachment to their ideas, creatively deviant employees are likely to explore new directions freely and to persevere against adversity in the creative process, allowing their creativity to flourish (Mainemelis, 2010). Thus, creative deviance leads to better creative performance and innovation by the focal employee.

Finally, creative deviance enhances challenge stressors for the focal employee. Challenge stressors (e.g., resource shortage, workload, and others) represent obstacles that employees need to overcome in order to learn, develop and perform well. On the other hand, hindrance stressors (e.g., organization politics, red tapeism, and others) are sources of frustration and are detrimental to work motivation and growth (LePine et al., 2005). Challenge stressors are positively associated with achievement motivation and job satisfaction (Cavanaugh et al., 2000). Creative deviance can lead to resource shortage, because the feedback and support from the supervisor are critical resources for employees (Ashford, 1986), which are not available due to the employee’s creative deviant behavior. We argue that this resource shortage can act as a challenge stressor that has a positive influence on work motivation (LePine et al., 2005), leading to better creative performance. Specifically, the intrinsically motivated nature of the creative deviance is likely to lead employees to transform resource shortages into positive challenges (Amabile et al., 1996) and search for more original solutions in unexpected and unconventional ways (Mainemelis, 2010).

Proposition 2a: Employees’ creative deviance is positively associated with focal employee’s creative performance and innovation.

### Creative Deviance and Negative Outcomes

Conventional wisdom would suggest that if an employee devotes time and effort toward developing a “bad” or un-useful idea (which he or she mistakenly assumed to be a good idea), he or she will waste time and resources (lab equipment, materials, software). Robinson and Bennett (1995) categorized the “wastage of resources” as the significant outcome of deviant workplace behavior. Wastage of resources is harmful for the organization and hinders organizational performance. Hollinger and Clark (1982) categorized “wastage of resources” as production deviance and defined it as the result of the violation of prescribed norms delineating the minimal quality and quantity of work. Mainemelis (2010) asserted a positive relationship between the creative deviance and wastage of resources. There are multiple reasons which can explain the positive link between creative deviance and the wastage of resources.

The first reason is the low base rate of successful implementation of the deviant creative idea. Mainemelis (2010) argued that creative deviance is more likely to occur when the idea is radical and defies existing norms. The extant research suggests that the risk of failure increases with an increase in the radicality of an idea (Stringer, 2000; Mainemelis, 2010; Alexander and Van Knippenberg, 2014). The greater the radicality of an idea, the more likely the idea developed will be rejected by management (March, 1991; Dewett, 2006; Baer, 2007). Since creative deviance is often associated with radical ideas – which are associated with high risk for failure – the employee likely invests the organizational resources like material, man-hours, etc., into a radical idea that will eventually fail. Thus, creative deviance would lead to the wastage of resources.

The second reason is the escalation of deviance (Fleming and Zyglidopoulos, 2008; Kelly et al., 2017) which means that one deviant act can give rise to another more serious deviant act. Creative deviance can engender a perception in the focal employee that he/she can break the rules of the organization with impunity. Such a perception is likely to develop after creative deviance because the stakeholders/beneficiaries involved in the creative project might provide tacit support to the focal individual when he/she decides to act against the mandate of the supervisor. Because of above perception, focal employee can break the organizational rules and norms, which ensure good quality work, leading to the wastage of resources (Hollinger and Clark, 1982). Escalation of deviance has previously been studied in social context (Fleming and Zyglidopoulos, 2008; Kelly et al., 2017). Escalation of deviance can occur in the workplace as well, where a small deviant behavior (for example, the neglect of supervisory mandate for creative idea) can lead to harmful behaviors, such as wastage of resources (production deviance).

The third reason is halt of resource exchange (Foa and Foa, 1980) between the focal employee and organization. When an individual decides to creatively deviate, he/she stops exchanging social resources, and does not receive supervisory feedback or top management support within the organization. Thus, the employee does not feel responsible for the commitment to quality work which is the return resource from the employee to the organization. Resource exchange has been regarded as extremely essential for creative performance and innovation in organizations (Wong et al., 2007). Creative deviance leads to the impediment of this essential part of the process of innovation and creative performance, thus generating the resource wastage due to unsuccessful implementation of ideas. In cases of creative deviance, the focal employee would be neglectful in the proper utilization of organizational resources (which he/she illegitimately exploit to pursue the rejected idea) as he/she will not feel responsible toward the delivery of quality work to the organization.

The above arguments suggest that it is highly likely that creative deviance can result in wastage of organizational resources. Apart from the wastage of organizational resources, creative deviance can also result in the deterioration of the focal employee’s emotional resources, for example, the relationship with the supervisor. Employees’ sense of managerial neglect of the mandate to act creatively and with autonomy can lead to the reduction of both employees’ trust and perspective of high-quality connections in their relationship with the leader. From the leaders’ perspective, creative deviance can also limit supervisors’ trust in the follower and can even lead to supervisors’ perception of resistance and subversion from the employee. These mutual dynamics can deteriorate the LMX relationship. LMX represents the relationship between supervisor and employee and affects the instrumental (work-related ties) and expressive (emotional ties) relationships between the focal employee and his or her supervisor (Graen and Uhl-Bien, 1995). We argue that once the supervisor limits the employee’s creativity, resulting in an employee enacting creative deviance, this can contribute to a negative mutual perspective. It can also reduce the level of open communication, leading to lower levels of LMX and limiting the emotional and relational resources.

Creative deviance by the focal employee might also pose an identity or ego-threat to the supervisor. In the extant literature, *ego threat* is defined as the threat to one’s self-esteem or self-image, as well as the threat to one’s public image and reputation (Baumeister and Boden, 1998; Leary et al., 2009). When the focal employee neglects the mandate from the supervisor, it creates a threat to the supervisor’s self-esteem and reputation. This ego threat to the supervisor can further deteriorate the leader-follower relationship (Kiewitz et al., 2016). Thus, we propose that:

Proposition 2b: Employees’ creative deviance leads to negative outcomes, such as wastage of resources and the deterioration of leader-member exchange relationships (limiting emotional and relational resources).

## Moderators of the Relationship Between Creative Deviance and Outcomes

The argument that creative deviance is a double-edged sword and can be a mixed blessing – due to the tension between the creative component and the deviant component (Mainemelis, 2010) – raises the questions of when will creative deviance produce positive outcomes, and when will it lead to negative ones. We suggest boundary conditions that moderate the relationship between creative deviance and its outcomes. Specifically, we apply the interactionist perspective of creativity (Woodman and Schoenfeldt, 1990; Woodman et al., 1993) to argue that the outcomes of creative deviance are guided by the interaction between the individual and various factors at different levels of the organization.

We included the moderators in our model based on four criteria. First, the moderators should be supported by the “interactionist perspective of creativity” (Woodman and Schoenfeldt, 1990; Woodman et al., 1993). Second, there must be some empirical support in the existing literature about these moderators impacting creative processes. Third, moderators should not be highly correlated with each other as it might reduce the explanatory power of our model. Finally, we wanted to represent four different levels of analyses and influence: (i) individual, (ii) team, (iii) organizational, and (iv) wider societal/environmental factors- which impact the relationship between creative deviance and its outcomes. The multilevel interaction is well supported by the interactionist perspective of creativity (Woodman et al., 1993). We further explicate below.

## Individual Level Moderators

### Perspective-Taking

Perspective-taking is defined as the process of imagining the world from the point of view of others, and understanding the desires, values, and choices of those others (Parker and Axtell, 2001; Galinsky et al., 2005). Extant literature is replete with research suggesting that perspective-taking reduces many cognitive biases in professional setups. For example, perspective-taking has a negative relationship with confirmation bias (Galinsky, 1999), reduces the power of stereotyping on individuals (Galinsky and Moskowitz, 2000) and develops a holistic perspective in individuals, helping them understand the deep roots of situational factors for a particular behavior (Boland and Tenkasi, 1995; Epley et al., 2004). Perspective-taking helps build social ties, which can be utilized by individuals to generate favorable outcomes in professional activities such as negotiations (Galinsky and Mussweiler, 2001).

Perspective-taking is directly linked with improvement in the quality of ideas. Mohrman et al. (2001) discovered that when creative academicians took the viewpoints of experts, the practical contribution of their research improved significantly more than their contribution without perspective-taking. Grant and Berry (2011) found that perspective-taking enhances the effect of intrinsic motivation on creative performance. The earlier research has also shown that perspective-taking increases the probability of the conversion of creative ideas into more useful products. This conversion occurs because perspective-taking enables the unbiased selection of good creative ideas and rejection of bad ideas (Dougherty, 1992; Purser et al., 1992). Because perspective taking facilitates better idea selection and idea development, it will lead to a stronger relationship between creative deviance and creative performance.

At the point when employees take the viewpoints of others, they are bound to think in an integrative style to solidify and adjust their perspectives (De Dreu et al., 2000). We suggest that attentive consideration of the points of view of others will provide employees with a standard for deciding which novel ideas are valuable and worth pursuing, versus those which are less useful and therefore disposable. When employees are instructed to stop developing an idea by their managers, they are less likely to gain the input of the manager or other stakeholders within the organization on the idea that they continue to develop at their discretion. Thus, if employees have a strong ability to gauge the perspective of the clients or the targets of the new product, the creative deviance can lead to better innovation. When employees can take the perspective of other team and organizational members who have a role in the process of turning the idea into a product (for example, employees from marketing, finance, management), idea development becomes more effortless. Employees need not talk to those organizational members directly about the product. Rather, a latent grasping of their opinion will make employees more able to develop an idea, allowing it to be accepted and pass the initial scrutiny, and have it become a product that can be utilized or implemented.

The motivational information processing theory supports our argument. As we mentioned earlier, motivational information processing theory (Kunda, 1990; Nickerson, 1998) suggests that individuals selectively process the information that is in harmony with their motivation, needs, and desires. Hence, it is highly probable that the perspectives of the peers and other stakeholders (like customers, vendors, etc.), who are interested in the creative idea of the focal employee would influence the thinking of the focal employee engaged in creative deviance.

On the one hand, when employees are high on perspective taking, they think about the positive, yet diverse viewpoints and opinions and they gain a more profound comprehension of how to make improvements to their creative ideas. This can generate more support and acceptance toward their ideas (Amabile et al., 1996). Thus, perspective taking helps an individual, engaged in creative deviance, to gather the necessary support, effectively assembled from various stakeholders (e.g., peers, other role holders in the organization, customers, etc.). This support from other stakeholders increases the likelihood of better creative performance and innovation by the focal employee. Thus, at high levels of perspective taking, the relationship between creative deviance and the positive outcome of creative performance is likely to be enhanced.

On the other hand, when employees are low on perspective taking, they are less likely to understand the needs, wishes, emotions, attitudes, and behaviors of other stakeholders within the organization. This may lead to a situation in which they will exploit organizational resources (time, talent, money, etc.) to develop an idea that is more likely to be rejected by others, at a later stage, thus leading to the resource wastage. Therefore, at low levels of perspective taking, we anticipate a stronger relationship between creative deviance and the negative outcome of wastage of resources.

We further argue that perspective-taking can also help in improving relationship with the supervisor. The first reason for our argument is that perspective-taking helps the focal employee improve the idea until it becomes acceptable per the standards of the supervisor. Thus, it is less likely that the supervisor will have a negative opinion about the work of the focal employee when the employee is high on perspective-taking. The second reason for perspective-taking positively influencing the relationship between the supervisor and the focal employee is that perspective-taking generates the other-orientation (inclination to consider the opinion of others) amongst the creative employees (Polman and Emich, 2011). Thus, employees high on perspective-taking would consider the opinion and judgment of the supervisor and listen to him/her more carefully. This focused attention on the supervisor’s instructions and views (though the supervisor rejected the idea) would create better communication between the focal employee and supervisor, and thus would lead to better LMX.

Proposition 3: Perspective-taking (individual level) will moderate the relationship between the creative deviance and its’ outcomes, such that the relationship between the creative deviance and the positive outcomes will be strengthened, and relationship with negative outcomes will be weakened, in the case of a high level of perspective-taking.

### Social Skills

Social Skills as a construct has been used as a broad concept in the extant literature, representing the social presence and the ability to influence social actors. There has been a lack of consensus on the precise definition of social skills amongst researchers (Riggio, 1986). However, we define social skills as the skillset of understanding others, understanding their intentions and choices accurately, communicating effectively, and persuading others to agree to one’s arguments (Cohen et al., 1986; Riggio, 1986). Riggio (1986) established the extensive construct of social skills including the following seven sub-factors of the broader concept: (a) emotional expressivity, (b) emotional sensitivity, (c) social expressivity, (d) social sensitivity, (e) emotional control, (f) social control, and (g) social manipulation.

*Emotional expressivity* represents the ability to express emotions, attitudes, and dominance. *Emotional sensitivity* represents the ability to receive and process in the right way the emotions, attitudes, and status expression from others. Similarly, *social expressivity* captures the skill of effective verbal communication influencing various social actors. *Social sensitivity* denotes a skillset of receiving and processing the messages from social actors and understanding the expressed concerns for social norms. On the control dimension, *emotional control* includes the skill to monitor one’s affect-laden communications and non-verbal cues. *Social control* represents the skill to effectively enact one’s role in the social fabric, regulate verbal communication, and present oneself effectively to the members of the social setup. *Social manipulation* represents the skills to manipulate social situations and social actors for one’s advantage. The social and emotional sensitivity dimensions of the social skills can enable a better understanding of the supervisor’s intentions even though a creative idea by the focal employee might have been rejected. Thus, an employee can make relevant changes in the idea (gauging the supervisor’s needs and concerns). At the same time, social and emotional expressivity dimensions would enable the employee to present the revised idea to the supervisor effectively and convincingly, which would increase the probability of a previously rejected idea of the focal employee’s being approved.

In a given social setup, social skills influence the choice of social roles, the membership of a social group, and the social behavior of an individual (McFall, 1982). Individuals with stronger social skills have more social-ties and are more powerful than others are in an organization’s social fabric (Friedman et al., 1980). The extant research has established that social skills are instrumental in gathering social support and in formation of friendship ties that lead to the flow of emotional resources toward the focal individual (Cohen et al., 1986). Emotional ties are a stronger predictor than instrumental ties of the positive work-related bonds between individuals (Casciaro and Lobo, 2008). Due to strong emotional ties, individuals with strong social skills will be more likely to convince others of the usefulness of their ideas and will, in turn, be able to gather the peer support necessary for the implementation of the idea. This will strengthen the chances of successful implementation of the creative idea by the focal individual involved in creative deviance.

In order to implement ideas that have been rejected by one’s supervisor, the employee may need the support of other stakeholders in the organization. Such support can aid the focal employee in various ways: for example, giving emotional, social, and financial support, other types of resources, and collaborations with others. Individuals who are involved in creative deviance, and have stronger social skills are more likely to form good relationships with their co-workers, team members, other managers and role holders in the organization (finance, HR, R&D, etc.). This will allow them to gather social support to develop an idea that is more likely to be brought to life and implemented, possibly positively contributing to the organization. However, employees with weak social skills would not be able to generate the support necessary for successful implementation of the idea, leading to lesser chances of their ideas developing into innovative products. Thus, in cases of weak social skills, the relationship between creative deviance and creative performance/innovation will be weaker.

Moreover, earlier studies have shown that individuals with good social skills can create and maintain strong interpersonal bonds with influential members of their social fabric (Kwan et al., 2008). Thus, emotional and social control embedded in the strong social skills will enable employees to manage the LMX better and avoid the communication errors that could spoil the relationship with a supervisor. Individuals with good social skills can manage the LMX better, thus causing low risk of LMX deterioration. Overall, the employee involved in creative deviance needs to have an entrepreneurial zeal for the successful implementation of the creative idea, in addition to an interpersonal orientation to maintain the harmonious relationship with the supervisor who gave the mandate to stop working on the deviant idea. The social skills would enable the focal employee to enhance both of the required qualities above (Baron and Markman, 2000), thus increasing the chances of better creative performance and decreasing the chances of spoiling a relationship with the supervisor. Hence, we propose the following.

Proposition 4: Social skills (individual level) will moderate the relationship between the creative deviance and its outcomes, such that in the case of strong social skills the relationship between the creative deviance and the positive outcomes will be strengthened and the relationship between the creative deviance and the negative outcomes will be weakened.

## Group Level Moderators

Next, we investigate the group-level variables that can influence the relationship between creative deviance and its outcomes. Little work has examined the team level contextual effects of creative deviance. There are three primary reasons behind our inclination to explore the *meso* moderators. First, the dynamics in a group can be very different from those on the individual level (e.g., team personality is different from the individual personality, Cable and Edwards, 2004; Humphrey et al., 2011) and therefore, the same variables at the group level can have very different work outcomes. Second, *Meso* thinking illuminates new processes and theories that are not visible at the individual level (Rousseau, 1985). Thus, theoretically sound research must investigate the group level phenomenon concerning work outcomes. Third, we wanted our research to be more relevant to practitioners. The contribution to practice is a function of the research’s potential to influence organizational outcomes. The linkage between individual variables (like creative deviance) and organizational level outcomes is of value to practitioners, and *meso* level thinking provides that linkage.

To enhance/reduce creative deviant behavior – which can have either a positive contribution to the organization or a negative one – practitioners should know which team level variables could influence those outcomes. Hence, *meso* thinking becomes very important. In the case of creative deviance, team-level processes and dynamics would influence the flow of emotional and instrumental resources, which might influence the relationship between creative deviance and its outcomes. In the upcoming section of group-level moderators, we investigate the moderating role of team-network structure and team climate for excellence in the relationship between creative deviance and its outcomes.

### Team Network Structure

The extant research has provided sufficient evidence showing that the team network structure influences the creative performance of team members (Guimera et al., 2005; Jia et al., 2014). Theoretically, the interactionist perspective of creativity suggests that the individual would interact within the group, and the team characteristics would influence his/her creative performance (Woodman et al., 1993; Anderson et al., 2014). We argue that the network structure of the group will determine the nature of this interaction, because the social support one gathers from the group depends on the group network structure (Borgatti et al., 1998; Morrison, 2002). In creative deviance, social support is especially important because it might rectify the shortage of supervisory support during the idea implementation process. The focal employee can access this social support through the group’s network structure (Perry-Smith and Mannucci, 2017). The type of network structure of the group can attenuate or accelerate this social support.

The type of social support required from the team is different for creative performance (idea generation and development) and innovation (idea implementation). At the idea generation and development stage, the focal employee would need acceptance and encouragement from his peers for the idea he/she is working on without supervisory support (Perry-Smith and Mannucci, 2017). The number of structural holes in the team’s network structure will facilitate the occupancy of the brokerage positions for the focal employee in the team’s network (Burt, 2001). Structural holes are a good source of influence and legitimacy. The team members traversing structural holes control the information flow and resource distribution between the unconnected nodes, and they can utilize this control to accumulate support for their creative ideas (Burt, 1992; Seibert et al., 2001). We argue that when the number of structural holes in the network is higher, it will be more likely that the employee engaged in creative deviance will occupy the brokerage position in the team’s network. He/she will be able to control the information and resources flow between the unconnected nodes in the team’s network structure.

Thus, the employee would have influence over the team, which would be easily convertible to social support for the creative idea being championed by the focal employee. Apart from influence for the focal employee, a network structure with more structural holes, will facilitate better vision and understanding of team members’ perspectives and opinions (Burt, 2004). Thus, the focal employee would be able to make the necessary improvements in the idea which can make it more suitable and acceptable to the team. Overall, more holes in the team network structure will make the selling and championing of the idea easier (Perry-Smith and Mannucci, 2017).

At the idea implementation stage, the network closure will be favorable for the innovation. At this stage, stronger resource exchange and common vision are required to develop the innovative product (Baer, 2012; Perry-Smith and Mannucci, 2017). We apply Coleman’s (1990) theory of network closure to argue that the network closure will facilitate strong connections for the focal employee, which in turn, can provide the necessary resources for idea implementation. The network closure facilitates the normative pressure of collaboration in the team (Coleman, 1990; Lingo and O’Mahony, 2010). Thus, the focal employee would be able to convince teammates to work closely on the idea implementation and generate an innovative product. Close networks also facilitate the information sharing among teammates (Burt, 2001) which is necessary in the idea implementation stage. Thus, a closed network structure will enable better chances for idea implementation.

The team network structure will also influence the relationship between creative deviance and the negative outcomes of wastage of resources and deterioration of leader member exchange. We argue that more structural holes will reduce the wastage of resources, whereas network closure will dampen the deterioration of leader member exchange. As outlined above, more structural holes in the team’s network will facilitate the occupancy of the brokerage positions by the focal employee who will have a better understanding of the opinions and suggestions of the team members. Thus, the focal employee would be able to make necessary changes to the idea, which would increase the likelihood of its success. When the focal employee is able to access teammates’ perspectives, he/she is able to give up the bad ideas and work on the better ideas, thus decreasing the likelihood of wastage of resources.

In contrast to open network (with more structural holes), the close network structure facilitates strong socio-economic bonds amongst teammates. Strong ties within the team facilitate the team identification of the team members (Guan et al., 2013). Thus, teammates will identify better with the team when the team network will be closed. Stronger identification with the team will facilitate positive exchange of emotions and ideas with the team leader as well. Thus, there will be stronger interpersonal bonds between the team leader and the employees in closed network structures. These interpersonal bonds will buffer the negative impact of the defiant behavior of the focal employee engaged in creative deviance. Hence, in the closed team networks, the negative relationship between creative deviance and leader-member exchange will be weaker.

Proposition 5a: The number of structural holes in the team’s network structure (team level) will moderate the relationship between creative deviance and its outcomes, such that in the case of more structural holes, the relationship between creative deviance and creative performance will be stronger and the relationship between creative deviance and wastage of resources will be weaker.
*Proposition 5b:* The n*etwork closure in the team’s network structure (team level) will moderate the relationship between creative deviance and its outcomes, such that in the case of higher network closure, the relationship between creative deviance and innovation will be stronger and the negative relationship between creative deviance and leader member exchange will be weaker.*

### Team Climate of Excellence

Organizational climate has been defined as a set of shared perceptions (of the policies, practices, and procedures that an organization rewards, supports, and expects) that is developed through group interaction (Schneider and Reichers, 1983; Kuenzi and Schminke, 2009). Because perceptions about climate can be diverse in different work units within a given organization, it can be defined and operationalized in workgroup settings (Kuenzi and Schminke, 2009). Accordingly, at the group level, the definition of *Team Climate for Excellence* is as follows: a set of shared group norms about “excellence of quality of task performance” in the group (West, 1990). Individual creativity is not particularly dependent on these norms; however, these norms represent a more general focus on the type of quality expected from creative performance (West, 1990; Anderson and West, 1998). Therefore, the team climate of excellence might not generate creativity among team members, but it will motivate them to develop and implement their creative ideas in such a way that upholds the high standards of expected excellent organizational performance - in our case, in the form of creative performance and innovation (Burningham and West, 1995).

The high-quality standards set for the team will lead to an increased focus by the employee on the implementation quality of his/her creative ideas. Good development and implementation of creative ideas would require constant quality control and interception by the supervisor. However, due to creative deviance, the support of a supervisor is not available to the focal employee, meaning that the quality control of interventions by a supervisor is not available. Hence, when creative deviance occurs, the quality of the creative idea’s implementation will depend on the strength of the team climate of excellence. Such a climate leads to better creative performance, possibly substituting the guidance and approval of the leader with the guidance of quality norms.

Employees within a team, which strives for excellent outcomes, are likely to have more resources available to develop their products and to evaluate them by testing if they meet the requirements of excellence. This is because of the higher team-level standards that shape their ethics within the workplace and what it means to have an excellent product. They are also able to gain better feedback from team members (co-workers) on how to develop an excellent creative product, possibly allowing for better use of the organizational resources available to develop previously rejected ideas into a creative product that can be useful to the organization (Wooten and Ulrich, 2017). Our argument is supported by the extant literature. For instance, Eisenbeiss et al. (2008) have found empirical support for the moderating role of team climate of excellence in the relationship between support for innovation and team innovative performance. The authors found that at lower levels of team climate of excellence, the support for innovation actually renders poor innovative performance from the team. Similarly, Anderson, and West (1998) showed that a climate of excellence predicts administrative efficiency and effectiveness of creative performance.

Team climate of excellence is strongly correlated with constructive controversy (Tjosvold, 1982; Anderson and West, 1998). Constructive controversy represents constructive criticism by team members when a focal employee does not follow set performance standards (Anderson and West, 1998). We argue that in cases when the implementation of an idea by the focal employee has the potential to waste team or organizational resources, the respective team members will criticize the idea and even suggest relevant changes needed to avoid the wastage of resources. Positive criticism from the teammates will decrease the likelihood of errors while implementing the creative idea. When supervisory support is available, constructive criticism from a supervisor reduces the chances of error (Baron, 1988). However, in cases of creative deviance, the implementation of creative ideas sustains risks of large amounts of error because supervisory support is not available. When a stronger climate of excellence is present, team members will provide the required constructive criticism to avoid errors in implementation (Koballa, 1992), and to minimize the chances of resource waste. Moreover, the criticism of peers would lead to better evaluation of creative ideas by the focal individual, prompting engagement in creative deviance in order to pursue the ideas that have genuine potential of manifestation in terms of innovation.

Team climate of excellence also leads to the better alignment of performance goals between supervisor and subordinates, which leads to better affective ties between the leader and follower (Pirola-Merlo et al., 2002). These affective ties between subordinate and leader would cushion the pressure building on the LMX due to creative deviance. Moreover, the team climate of excellence would reduce the probability of relationship deterioration between the team members and the leaders by enhancing the possibility of good innovative performance (which will be appreciated by the leader). In most teams, the goals of supervisors and employees are interdependent, and the achievement of individual task goals by employees leads to better LMX (Martin et al., 2016). In stronger climates of excellence, the resolution of individual performance goals will enable achievement of the performance goals set by supervisors. When a focal individual in such a team engages in creative deviance, the likelihood of a poor LMX will be minimized.

Proposition 6: Team climate for excellence (team level) will moderate the relationship between creative deviance and its’ outcomes such that at higher levels of the climate of excellence the relationship between the creative deviance and positive outcomes will be strengthened and the relationship between the creative deviance and negative outcomes will be weakened.

## Organizational Level Moderator

Next, we explore the organizational level characteristics, which influence the relationship between creative deviance and its outcomes. The rationale for investigating the organizational level moderators lies in the fact that individual level relationships (e.g., between creative deviance and creative performance) change due to changes at an organizational level. The systems and processes at the organizational level constitute important contextual factors that affect individual level relationships. Thus, the multilevel models must look at organizational level factors to outline the contexts that influence individual level relationships (Rousseau, 1985). Apart from this, earlier researchers suggest that each organization in the contemporary business world is going through a continual phenomenon of change, which affects internal processes at all levels, including relationships at the individual level (Daft, 1998). Hence, from practitioners’ point of view, it is essential to outline the phenomenon at the organizational level – which bares influence on important relationships at the employee level. For example, organizational context influences the leadership style of supervisors and can influence LMX (Kark and Van Dijk, 2007). The relationship between creative deviance and its outcomes, such as creative performance and change in LMX, is bound to be influenced by the organizational context. Accordingly, in our theoretical framework, we investigate the role of organizational structure as an organizational level factor influencing the relationship between creative deviance and its outcomes.

### Organizational Structure

The intermittent set of relationships amongst the members of an organization constitute the organizational structure (Donaldson, 1996). These relationships include the following: (a) relationships of authority; (b) relationships of hierarchy or reporting; (c) desired behaviors as per the organizational policy; (d) decision making norms, e.g., centralization; and (e) communication norms. Many other behavioral norms and rules fall under the gamut of the organizational structure. The formal parts of organizational structure are well defined and written (for example, a and b in the above list), and there can be informal parts which are not documented and open to interpretation, such as behavior norms (Pennings, 1992; Donaldson, 1996). Organizational structure can be bifurcated at the macro level into the *mechanistic* and *organic* forms of the organizational structure (Burns and Stalker, 1961).

Burns and Stalker (1961) succinctly delineated the characteristics of these two forms. A mechanistic organizational structure is characterized by (a) differentiation of the tasks and functions due to differentiation in placement; (b) strict definitions of job functions; (c) precise definitions of hierarchy levels; (d) well-defined boundaries of rights, responsibilities, and methods of each functional role; and (e) hierarchical structure of control, authority and communication. In contrast, the organic structures are characterized by (a) contribution of special skills and knowledge of each member to the common task of concern; (b) much overlap amongst functions; (c) flexibility in role definitions, depending on the nature of the task; (d) blurred lines in terms of rights and responsibilities assigned to specific positions; and (e) no technical definition of the task limited to a particular method, thus enhancing the possibility of innovation.

In the mechanistic structures, supervisors are in positions that are substantially more powerful, and employees might face severe repercussions if they defy any form of managerial order (Aryee et al., 2008). In the organizations or departments with a mechanistic structure, the supervisor will have enough power to thwart the creative intentions of subordinates who have defied orders. In such cases, the negative reactions of the supervisor will discourage the successful implementation of a creative solution, and creative deviance will therefore be less likely to produce positive outcomes.

In organic structures, there are fewer power imbalances, and decision-making processes are decentralized. These decentralized decision-making processes create the opportunity for employees to behave in a more innovative manner while solving problems in focal projects and tasks (Ashforth, 1994; Donaldson, 2000). A plethora of research studies has found that an organic structure fosters innovation and creativity (e.g., Pierce and Delbecq, 1977; Meadows, 1980; Hull and Hage, 1982). The most prominent argument for the relationship between organic structure and innovation in the literature is that decision-making is much more decentralized in organic structures, and employees have more autonomy to implement creative ideas in decentralized structures successfully. When creative deviance occurs, the organic structure will facilitate decentralized decision making that will empower the employees to take the initiative on the creative project – against the bosses’ will - as well as pool in the collective skills and resources to generate good innovation. Thus, creative deviance is more likely to generate good creative performance in organic structures.

Since communicative and behavioral norms are parts of the organizational structure, the relationship between LMX and the organizational structure is inherent and intuitive. The mechanistic structure has been established, in the extant literature, as a source of considerable power imbalance between the leader and member, as well as a boundary condition enhancing the negative effects on LMX (see for example, Aryee et al., 2008; Qian et al., 2017). In creative deviance, the employee’s neglect of a supervisor’s mandate would trigger the supervisor’s dissatisfaction; if the supervisor has sufficiently more power in the structure, he/she will express that dissatisfaction in the LMX with impunity. In a mechanistic structure, the negative relationship between the creative deviance and LMX will, therefore, be stronger.

The organic structure, on the other hand, has proven to strengthen the relationship between positive LMX and innovation (Pan et al., 2012). It facilitates the decentralized decision-making in the team when the supervisor is expected to be more tolerant of the opinions and ideas of the employees. With creative deviance, the decision of an employee to go against the mandate of a supervisor – with intent to innovate – is less likely to be construed in a negative light by the supervisor. Thus, in an organic structure, the negative relationship between creative deviance and LMX will be weaker.

Proposition 7: Organizational structure (organizational or business unit level) will moderate the relationship between creative deviance and its outcomes. The mechanistic structure will strengthen the relationship between creative deviance and negative outcomes, while organic structure will strengthen the relationship between creative deviance and positive outcomes.

## External Environment Level Moderator-Uncertainty

The most important factor of innovation in any organization is the environment of the firm (Donaldson, 1996). The competition a firm faces from other organizations leads to the focal firm’s attempts at innovation in order to maintain a competitive advantage. The change in the external environment creates a demand for innovation in the internal systems because the existing systems are unfit to keep up with the change in environment on varying occasions (Scott, 1961). With creative deviance, the same environment can either facilitate or thwart the development of the creative products. Understanding the existing literature, we envisage the role of uncertainty in the external environment as a contextual factor which influences the relationship between creative deviance and its outcomes.

### External Environment Level Moderator-Uncertainty

The extant literature has shown that uncertainty in the external environment of the firm facilitates innovation on existing projects and processes within the firm (see, for example, Özsomer et al., 1997; Freel, 2005; Wang et al., 2011). Environmental uncertainty demands a change within internal systems, processes, and project methodologies. Such a change is possible only when the existing way of thinking is challenged. The internal characteristics of organizations should be in harmony with the external pressure in order to innovate and change. In such a scenario, the “upper echelons” of the organizations, who are responsible for developing an organizational strategy that suits the external environment (Hambrick and Mason, 1984), support innovation and radical changes in internal systems. Creative deviance is one such phenomenon that facilitates radical change (Mainemelis, 2010). Creative deviance has an inherent characteristic to change the way of thinking and to challenge the existing norms and *status quo*. Thus, in an uncertain external environment of the firm, top management would see creative deviance in more favorable light.

Moreover, creative deviance is fostered when the focal organization puts more emphasis on creativity than conformity (Mainemelis, 2010). An external-environmental context of high uncertainty is likely to force an organization to prioritize creativity over conformity as the innovation can create a buffer from organizational uncertainty (Shane, 1995). In such situations, the organization is likely to allow for the use of more resources to search for novel ways of doing things. The entrepreneurs, for instance, utilize maximum resources to innovate when environmental uncertainty is high (York and Venkataraman, 2010). The creative deviants – to produce better creative performance – can then utilize these resources. Thus, creative deviance is more likely to produce innovative products and services when there is uncertainty in the external environment.

Uncertainty in the external environment also changes the focus of top management toward innovation (Russell and Russell, 1992). Top management is more open to changing the strategy that suits the external environment’s demand to be flexible and adaptive to market demands. For example, the strategy of “disruptive innovation” is one that is used to suit the rapidly changing customer demands in the market (Christensen et al., 2015). We argue that the strong focus of top management on innovation would make them tolerant toward the risk of failure associated with creative deviance. Thus, they will support creative deviants directly or indirectly in order to gain an innovation breakthrough. The support from top management would let the focal employee get enough power so that, in spite of creatively deviating, he/she would be able to bargain his/her terms with the corresponding supervisor.

Further, aligning themselves with the goals of the top management, supervisors would avoid viewing the creative deviance negatively. Due to changing goals, supervisors would rather change the formal/informal reward systems to increase the zeal for innovation in the respective teams, and to trigger voluntary out-of-the-box thinking (Lovibond and Colagiuri, 2013). The supervisors’ appreciation of the pro-active behavior toward innovation will actually foster the LMX with the employees engaged in creative deviance. Thus, it is less likely that creative deviance will lead to bad LMX.

Proposition 8: Uncertainty in the external environment (external environment level) will moderate the relationship between creative deviance and its outcomes such that at higher levels of uncertainty the relationship between creative deviance and positive outcomes will be stronger and the relationship between creative deviance and negative outcomes will be weaker.

## Discussion

The phenomenon of creative deviance has been ignored in the existing literature, and our research attempts to fill this literature gap. We expand the theoretical nomological network of creative deviance and theoretically delineate the multilevel boundary conditions, which can influence the relationship between creative deviance and its outcomes. More importantly, we investigate how creative deviance can act as a mechanism, leading to varying possible positive and negative consequences of prosocial motivation. We theoretically establish that prosocial motivation can lead to deviant behavior (i.e., creative deviance), and that creative deviance, in turn, can lead to both positive and negative organizational outcomes. Specifically, we propose that creative performance and innovation are the positive outcomes of creative deviance, whereas wastage of resources and deterioration of LMX are the negative outcomes of creative deviance.

We further explore boundary conditions of the relationship between creative deviance and the outcomes at four different levels of analyses, in order to determine when creative deviance is likely to lead to positive outcomes, and when it is likely to lead to negative ones. Perspective-taking and social skills moderated the above relationship at the individual level, team network structure and team climate of excellence were the boundary conditions at the group/team level, and the organizational structure was investigated as an organization level moderator. Further, we focus on the role of uncertainty as the external environment level factor that influences the interaction between creative deviance and its outcomes.

Our multi-level model addresses the call of earlier researchers (see, for example, Rousseau, 1985; Hitt et al., 2007) to engender the multi-level thinking within robust theory development of nascent constructs – such as creative deviance. Our model also addresses the theme of this issue, which calls for the investigation of the possible negative outcomes of positive prosocial motivation. We propose a conceptual framework through the examination of the construct of creative deviance, which holds the positive aspect of “creativity” and the potentially negative aspect of “deviance.” Our framework illustrates the dual (positive and negative) outcomes of creative deviance and its moderators (i.e., boundary conditions). Thus, our framework can comprehensively answer the questions of why, how and when prosocial motivation can lead to negative outcomes.

The counter intuitive appeal of the current topic lies in the fact that the existing literature is majorly inclined toward the positive effects of prosocial behavior (cf. Coyne, 2013), while the current theme encourages researchers to explore the negative side of prosocial behavior.

In attempting to uncover how theory and research can be exciting, meaningful and interesting, Davis (1971) suggested exploring theories and actual situations that are surprising, by tracking how a negative or “bad” process or phenomenon can be transformed into a “good” or a positive one. Our research is in line with this direction; however we show when “aiming for good can be bad,” building on and attempting to understand situations and dynamics in which attempting to bring good (behaving in a prosocial manner), can be harmful.

Our research contributes to several different areas of literature. The foremost contribution of our research is to the rather scant literature on creative deviance. Mainemelis (2010) introduced the concept of creative deviance, and since then, there has been almost negligible research on this interesting construct (cf. Lin et al., 2016). One of the reasons may be the nascent stage of theory development for this novel construct. By expanding the theoretical nomological network for this construct, we have tried to fill this literature gap. Our model shows how creative deviance interacts with context, and what can be the major positive as well as negative outcomes of creative deviance. Our conceptual model can contribute to the inception of more interesting and theoretically sound ideas for the empirical investigation of the antecedents and consequences of this relatively new construct, enabling a wider understating of how it can be played out in interaction with variables at multiple levels of analyses.

The second contribution of our research is to the literature on prosocial behavior and prosocial motivation (Eisenberg and Fabes, 1991). The prosocial intentions have been related to constructive deviance (Warren, 2003; Vadera et al., 2013). This is the first study linking prosocial motivation to creative deviance. Creative deviance is theoretically separate from constructive and destructive deviance. Destructive deviance refers to norm breaking to harm the organization and constructive deviance refers to norm-breaking to conform to hyper norms of social benefit (Warren, 2003; Galperin, 2012). Distinct from the above forms of norm violation, creative deviance is a specific case of breaking the norm of following one’s supervisor’s mandate with creative intentions in mind. Such intentions can lead to both positive and negative outcomes for the organization (Mainemelis, 2010). We assert that all three type of deviance – destructive, constructive, and creative – fall under the broad umbrella of norm-violating behavior, and that prosocial motivation can be an antecedent to constructive and creative deviance.

The third contribution of our research is to the literature on creativity. Our paper suggests that when an individual goes against the mandate of a supervisor to implement a creative idea, it can lead to either positive outcomes of creative performance and innovative products, or negative outcomes like wastage of resources and compromised LMX. The concept of creative deviance itself connects the literature of creative thinking and deviant behavior. It suggests that the deviant behavior of neglecting the mandate of a supervisor can produce positive outcomes – provided there is creative potential in the idea, and contextual factors (at individual, team and organizational levels) supporting the implementation are present. Thus, we delineate how creativity can be enhanced in different conditions.

## Limitations and Future Research

Although our paper contributes to multiple areas of literature, it has some noteworthy limitations. The first limitation is the focus on prosocial motivation’s ability to enhance creative deviance; we do not know how common it is that prosocial motivation may lead to creative deviance. Furthermore, the strength of the relationship between prosocial motivation and creative deviance is not clear. For example, in a certain context, people who are prosocial can actually show low creative deviance.

Second, although we develop a broad multi-level model of moderators, we focus on specific variables. We chose these variables specifically to represent the different levels – as exemplary moderators – however, there are other variables we could have considered in each level as well. For instance, at the individual level openness to experience, extroversion and narcissism can interact with creative deviance to either intensify (openness and extroversion) or attenuate (narcissism) the effect of creative deviance on positive outcomes. At the team level, conflict can interact with creative deviance to strengthen or weaken the relationship with creative outcomes. Similarly, the level of emergency and crisis in the organizational-level operations might demand creative deviance to provide much needed innovative solutions.

Third, in this paper, our moderators are based on the interactionist perspective of creativity (Woodman and Schoenfeldt, 1990). There are many other theories which could explain other boundary conditions. For example, the theory of reactance (Brehm, 1966) could explain why autocratic leadership will act as a boundary condition for creative deviance. Although we have clearly defined our criteria for moderators (see the section on boundary conditions), this paper is limited such that it does not explore all possible moderators. Integration of multiple (more than two or three) theories and much expanded scope and breadth was required to add more moderators, which could have been a tradeoff with clarity, accuracy, and focus. However, the non-inclusion of all possible boundary conditions remains a limitation of this paper. We encourage future researchers to explore more moderators in our model.

The fourth limitation is our focus solely on prosocial motivation as the source of creative deviance. Individuals can become involved in creative deviance in order to gain personal benefits, personal intrinsic rewards, or due to other pro-self motives. In this paper, due to our interest in prosocial behaviors, we do not develop these other directions. We strongly encourage future researchers to delineate a separate model of pro-self motivation leading to various outcomes through creative deviance and explore its boundary conditions.

Finally, we have not explored organizational culture as a boundary condition. Organizational culture and overall society culture (tight vs. loose cultures) can impact the relationship between prosocial motivation and creative deviance, as well as the relationship between creative deviance and its outcomes. The construct of tightness relates to cultures that have strong norms, and little tolerance for deviance, versus lose cultures that have weak norms and high tolerance for deviance (e.g., Gelfand et al., 2006, 2011; Harrington and Gelfand, 2014; Roos et al., 2015). Based on this, it is possible that in tight cultures, people will tend to enact less creative deviance, as a form of prosocial behavior, in comparison to looser cultures. These limitations are possible directions for future investigation.

While our model has some limitations, it provides a fertile source of ideas ripe for empirical research which can enrich the field of creative deviance and test the counterintuitive appeal of prosocial motivation leading to negative outcomes. It can also enable scholars to test the mixed blessing of creative deviance and the different conditions that determine those mixed blessings. We encourage future research to empirically examine the conceptual underpinnings of our model, as well as any related directions. We recommend the mixed design method for testing our model. Experimental design can provide internal validity to such research, while a field study can ensure its external validity. The experiment may be based on scenarios, vignettes, or computer-based interventions where the participants can be given an opportunity to go ahead with their creative idea against the will of their supervisors. The role of the supervisor can be manipulated in the experiment. Other various moderators as well can be manipulated in the scenario or vignette. In a field study, researchers can use the standardized scales of prosocial motivation (Grant, 2008), creative deviance (Lin et al., 2016), and LMX (Graen and Uhl-Bien, 1995). It is important that future studies measure creative performance and wastage of resources through supervisor reports in order to avoid self-rating bias and common method bias. Future research should also include participants from different cultures in order to test if cultural values can act as a moderator in the relationships depicted by our model.

## Conclusion

To conclude, we offer a novel conceptual model in order to understand how deviant behavior in the form of creative deviance can be a manifestation of prosocial behavior, leading to both negative and unwanted organizational outcomes, as well as positive and productive outcomes. We show at multiple levels what can influence the relationship between creative deviance and its mixed (positive and negative) outcomes. Our paper will contribute to firms, allowing them to create the conditions in which prosocial enactments of creative deviance may result in positive, creative, and innovative outcomes.

## Author Contributions

All authors listed have made a substantial, direct and intellectual contribution to the work, and approved it for publication.

## Conflict of Interest

The authors declare that the research was conducted in the absence of any commercial or financial relationships that could be construed as a potential conflict of interest.
